# Altered gut microbiota and inflammatory cytokine responses in patients with Parkinson’s disease

**DOI:** 10.1186/s12974-019-1528-y

**Published:** 2019-06-27

**Authors:** Chin-Hsien Lin, Chieh-Chang Chen, Han-Lin Chiang, Jyh-Ming Liou, Chih-Min Chang, Tzu-Pin Lu, Eric Y. Chuang, Yi-Cheng Tai, Chieh Cheng, Han-Yi Lin, Ming-Shiang Wu

**Affiliations:** 1 Department of Neurology, National Taiwan University Hospital, College of Medicine, National Taiwan University, Taipei, 100 Taiwan; 2 Department of Gastroenterology, National Taiwan University Hospital, College of Medicine, National Taiwan University, Taipei, Taiwan; 3 Graduate Institute of Clinical Medicine, College of Medicine, National Taiwan University, Taipei, Taiwan; 4 Department of Neurology, Taipei Veterans General Hospital, Taipei, Taiwan; 5 Bioinformatics and Biostatistics Core, Center of Genomic and Precision Medicine, National Taiwan University, Taipei, Taiwan; 6 Institute of Epidemiology and Preventive Medicine, National Taiwan University, Taipei, Taiwan; 7 Graduate Institute of Biomedical Electronics and Bioinformatics, National Taiwan University, Taipei, Taiwan; 8 Bioinformatics and Biostatistics Core, Center of Genomic Medicine, National Taiwan University, Taipei, Taiwan; 9 Department of Neurology, E-Da Hospital, Kaohsiung, Taiwan

**Keywords:** Parkinson’s disease, Gut microbiome, Dysbiosis, Cytokines, Neuroinflammation

## Abstract

**Objective:**

Emerging evidence suggests that gut microbiome composition alterations affect neurodegeneration through neuroinflammation in the pathogenesis of Parkinson’s disease (PD). Here, we evaluate gut microbiota alterations and host cytokine responses in a population of Taiwanese patients with PD.

**Methods:**

Fecal microbiota communities from 80 patients with PD and 77 age and gender-matched controls were assessed by sequencing the V3–V4 region of the 16S ribosomal RNA gene. Diet and comorbidities were controlled in the analyses. Plasma concentrations of IL-1β, IL-2, IL-4, IL-6, IL-13, IL-18, GM-CSF, IFNγ, and TNFα were measured by a multiplex immunoassay and relationships between microbiota, clinical characteristics, and cytokine levels were analyzed in the PD group. We further examined the cytokine changes associated with the altered gut microbiota seen in patients with PD in another independent cohort of 120 PD patients and 120 controls.

**Results:**

Microbiota from patients with PD was altered relative to controls and dominated by *Verrucomicrobia*, *Mucispirillum*, *Porphyromonas*, *Lactobacillus*, and *Parabacteroides*. In contrast, *Prevotella* was more abundant in controls. The abundances of *Bacteroides* were more increased in patients with non-tremor PD subtype than patients with tremor subtype. *Bacteroides* abundance was correlated with motor symptom severity defined by UPDRS part III motor scores (rho = 0.637 [95% confidence interval 0.474 to 0.758], *P* < 0.01). Altered microbiota was correlated with plasma concentrations of IFNγ and TNFα. There was a correlation between *Bacteroides* and plasma level of TNFα (rho = 0.638 [95% CI: 0.102–0.887], *P* = 0.02); and a correlation between *Verrucomicrobia* abundance and plasma concentrations of IFNγ (rho = 0.545 [95% CI − 0.043–0.852], *P* = 0.05). The elevated plasma cytokine responses were confirmed in an additional independent 120 patients with PD and 120 controls (TNFα: PD vs. control 8.51 ± 4.63 pg/ml vs. 4.82 ± 2.23 pg/ml, *P* < 0.01; and IFNγ: PD vs. control: 38.45 ± 7.12 pg/ml vs. 32.79 ± 8.03 pg/ml, *P* = 0.03).

**Conclusions:**

This study reveals altered gut microbiota in PD and its correlation with clinical phenotypes and severity in our population. The altered plasma cytokine profiles associated with gut microbiome composition alterations suggest aberrant immune responses may contribute to inflammatory processes in PD.

**Electronic supplementary material:**

The online version of this article (10.1186/s12974-019-1528-y) contains supplementary material, which is available to authorized users.

## Introduction

Parkinson’s disease (PD) is a common neurodegenerative disorder and is caused by a combination of genetic and environmental risk factors [1]. Aggregations of intra-neuronal α-synuclein known as Lewy Bodies (LB) are pathological hallmarks of this disease. The enteric nervous system (ENS) is among the structures earliest affected by LB pathology in the disease process of PD [2]. Accordantly, gastrointestinal dysfunction, particularly constipation, is the most common non-motor PD symptom and often precedes the motor disability onset by decades [3].

Emerging evidence suggests that gut microbiota may act as environmental triggers to promote neuronal degeneration and motor dysfunction through microglial activation in α-synuclein-overexpressing mice [4]. These observations are consistent with the altered gut microbiomes reported in patients with PD [5–12], although the reported gut bacterial profiles are heterogeneous. These inconsistent results may be related to the variable geographic origins and ethnic backgrounds of PD patients included in previous studies. An epidemiology study has shown that the PD incidence and prevalence is lower in Asians than those in Western populations [13], and there is clear evidence for distinct genetic causes of PD between these two populations. For example, the frequency of the prevalent p.G2019S mutation in the leucine-rich repeat kinase 2 (*LRRK2*) gene accounts for up to 40% of PD cases in North African Arab-Berbers and Ashkenazi Jews but is found in less than 0.1% of Asian PD patients [14], suggesting that Asian patients with PD may have different gut microbiota than Western PD patients. Gut microbiome composition alterations are also linked to aberrant immune responses, which are often accompanied by abnormal production of inflammatory cytokines [15], reinforcing the role of neuroinflammation in PD. However, few studies have explored the gut microbiome and related host cytokine responses in patients with PD. Here, we characterize fecal microbiota and peripheral cytokine alterations in a Taiwanese PD population and analyze the relationships between changes of fecal microbiota and PD clinical characteristics.

## Methods

### Study participants

**Fig. 1:**
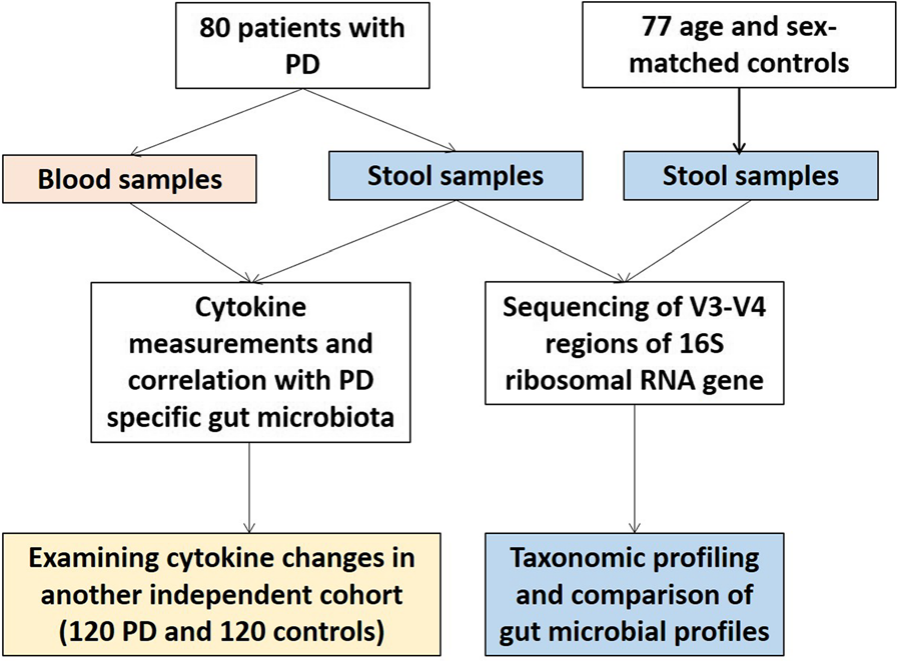
Flow chart and enrolled participants in the current study

The study flow chart was depicted in Fig. 1. A total of 397 study participants were included in this study, comprising 80 PD patients and 77 age and sex-matched control subjects without evidence of PD in the first part of the study to compare the gut microbiome changes, and an additional independent cohort of 120 patients with PD and 120 age and sex-matched controls were enrolled in the second part of the study to examine the cytokine changes associated with the altered gut microbiota seen in patients with PD. All participants provided informed consent and the study was approved by the institutional ethics board of National Taiwan University Hospital.

PD was diagnosed according to the United Kingdom PD Society Brain Bank clinical diagnostic criteria [16]. The motor phenotypes, i.e., tremor or non-tremor predominant type, were practically determined by the initially presenting symptoms of the patients, which was recorded in the medical records. Healthy control subjects, who were neurologically normal patients visiting our clinics, spouses, or accompanied friends of the PD patients, were recruited from the same institute. Participants with the previous diagnosis of inflammatory bowel disorders, irritable bowel syndrome, colitis, or colon cancer were excluded. Individuals currently taking antibiotics or probiotic supplements within 3 months of sample collection were also excluded. A comprehensive dietary history (Food Frequency Questionnaire) with 64 items [17] and medical comorbidities were collected.

### Evaluation of motor and non-motor symptoms

Motor symptom severity was evaluated using the motor sub-scores of the Unified Parkinson’s Disease Rating Scale (UPDRS part III) and Hoehn-Yahr staging, which were performed for each subject in both the practically defined “off” medication state (patients willing and able to withhold all PD medications for at least 12 h prior to assessment) and the “on” medication state (1 h after taking their usual morning PD medications that same day). Non-motor symptoms were assessed by the Non-Motor Symptom assessment Scale (NMSS), and cognition was examined with the Mini-Mental State Examination (MMSE) during the “on” phase of the PD patients. Constipation was diagnosed using the Rome III Criteria [18]. Furthermore, the severity of constipation was also assessed by a sub-score of the NMSS, which is defined as a frequency of defecation less than three times a week.

### Sample collection and DNA extraction

Stool samples were collected into stool specimen collection tubes containing DNA stabilizer (Sarstedt), which were immediately flash-frozen on dry ice, and stored at − 80 °C until analysis. Total fecal DNA was extracted using a QIAamp DNA Stool Mini Kit (Qiagen, Hilden, Germany) as previously described [8]. Blood samples of 10 ml of venous blood were drawn from each participant at enrolment and were centrifuged (2500 g for 15 min) within 1 h of collection. The plasma was aliquoted into cryotubes following centrifugation and stored at − 80 °C for cytokine analysis.

### 16S ribosomal RNA gene amplicon and sequencing

We used universal primers linked with indices and sequencing adaptors to amplify the V3–V4 regions of the bacterial 16S ribosomal RNA (16S rRNA) gene after extraction of total DNA from the fecal samples. The amplicons were sequenced on an Illumina Miseq platform to obtain 300-bp paired-end reads and for taxonomic assignment. Detailed descriptions of the amplicons and the sequencing analysis protocol are provided in the Additional file 1: Supplementary Methods.

### Measurement of plasma cytokine levels by a multiplex immunoassay

The ProcartaPlex Human Th1/Th2 Cytokine Panel 11plex (Thermo Fisher Scientific, Vienna, Austria) was used to measure plasma concentrations of IL-1β, IL-2, IL-4, IL-6, IL-13, IL-18, GM-CSF, IFNγ, and TNFα according to the manufacturer’s instructions.

### Statistical analyses

Comparisons between groups were performed with Student’s *t* tests and chi-square tests for quantitative and categorical variables, respectively. Variables that followed a Gaussian distribution were compared with two-tailed *t* tests or analysis of variance (ANOVA). We tested the homogeneity of variances by using Levene’s test. For variables that violated the assumptions of normality or homoscedasticity, the groups were compared with non-parametric Mann-Whitney *U* test (for two groups) or Kruskal-Wallis test (for more than two groups).

Differences were tested at the operational taxonomic units (OTUs), genus, and family levels. Taxa present in < 10% of samples were removed. The relative abundance of each taxon in PD patients versus controls, the Chao1 diversity index, and the Shannon entropy index were examined using the analysis of composition of microbiomes (ANCOM) and Kruskal-Wallis rank sum test, which both tests were conducted using default parameters in the python implementation of ANCOM in scikit-bio 0.4.2 (http://scikit-bio.org/docs/0.4.2/index.html) and the Kruskal-Wallis test was run using kruskal.test in R package. Correlations between plasma cytokine concentrations and genera expressed sequence counts with a prevalence > 10% were calculated using Spearman’s rank-correlation analysis under the assumptions that there is a non-linear relationship between the examined variables. The genera were then subjected to generalized linear model (GLM) analysis with a negative binomial distribution and controlling for zero-inflation as appropriate in the R package glmmADMB. Several factors were adjusted for different confounders including age, sex, and diet (the mean daily intake amount of protein, carbohydrates, total fat, and dietary fiber). Correlations between PD clinical parameters or plasma cytokine concentrations and genera expressed sequence counts with a prevalence > 10% were calculated using Spearman’s rank-correlation analysis for 80 PD patients under the assumptions that there is a non-linear relationship between the examined variables. All analyses were performed with Stata (StataCorp LP, College Station, USA) software and R software (version 3.1.0, the R Project for Statistical Computing). A *P* value < 0.05 was considered significant.

## Results

### Clinical characteristics of PD and control groups

The demographic and clinical information of 80 patients with PD and 77 control subjects enrolled in the first step of the study are summarized in Additional file 2: Table S1. A higher proportion of the PD group reported constipation than the controls.

(66.3% vs 12.3%, *P* < 0.01). The mean UPDRS part III scores and Hoehn-Yahr stages during the “on” and “off” state as well as NMSS scores are also included for patients with PD. There was no difference in age or sex between the two groups. There were more medical comorbidities including diabetes mellitus and hypertension in controls than in patients with PD. Of the clinical variables, the intensity of constipation was higher in PD patients with more advanced Hoehn-Yahr stages than in PD patients at early motor stages (*P* < 0.001 by one-way ANOVA).

### Patients with PD have altered and more diverse gut microbiota

**Fig. 2:**
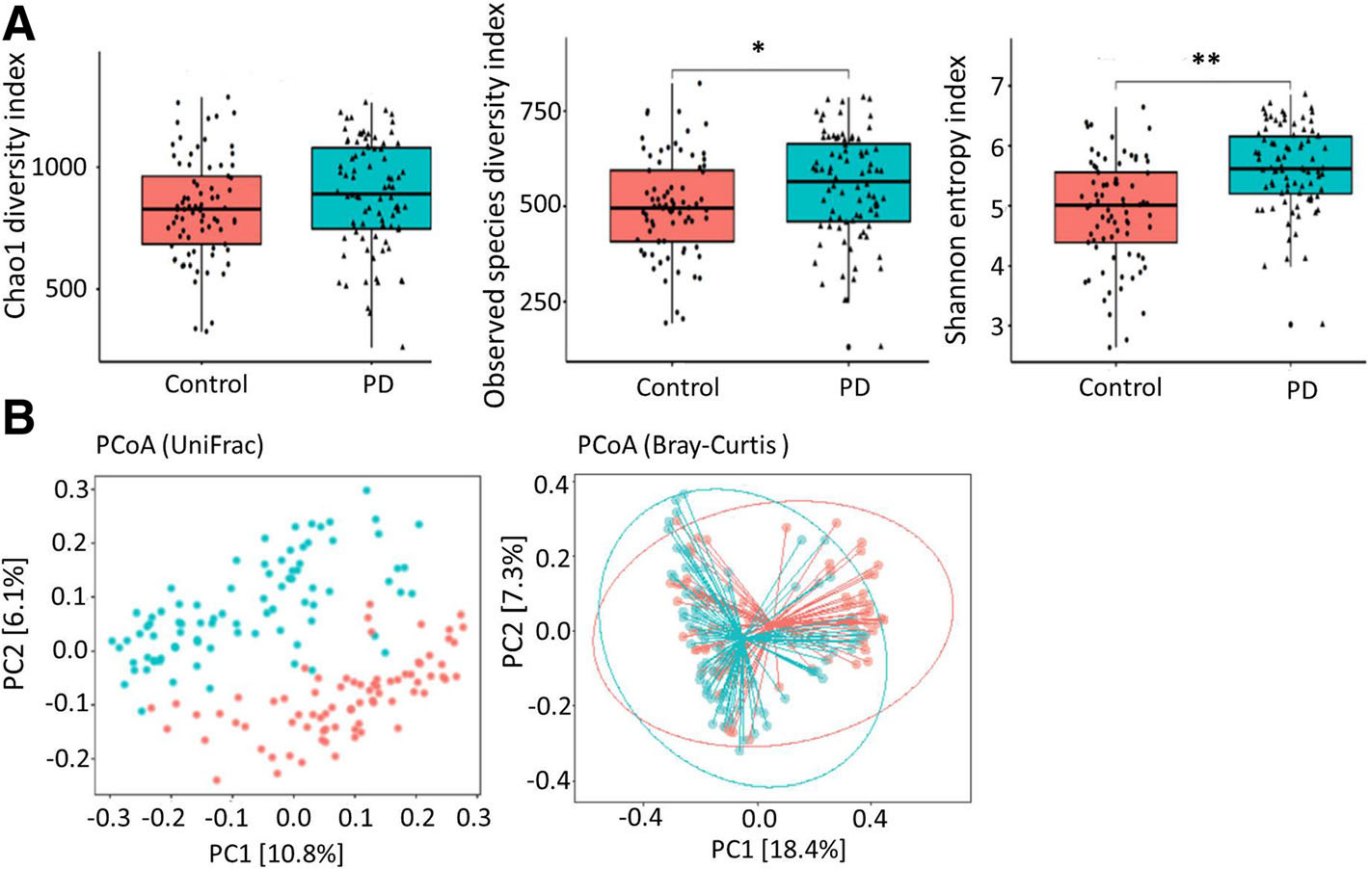
The α-diversity and β-diversity indices of the fecal microbiome in the PD and control groups. **a** Box plots depict differences in the fecal microbiome diversity indices between the PD and control groups according to the Chao 1 index, observed species index, and Shannon index based on OTU counts. Each box plot represents the median, interquartile range, minimum, and maximum values. OUT: operational taxonomic units (**b**) PCoA plots of bacterial β-diversity based on the weighted UniFrac distance (left panel) and Bray-Curtis dissimilarity (right panel) analyzed according to health status. Patients with PD and age-matched controls are colored in blue and red, respectively

We then characterized the bacterial gut microbiota associated with PD by high-throughput sequencing of the V3–V4 region of the 16S ribosomal RNA gene. We measured the bacterial richness within each sample from both the PD and control groups using three different methods, the observed number of operational taxonomic units (OTUs), the Chao1 diversity index, and the Shannon entropy index. The bacterial gut microbiota from patients with PD was more diverse than those from controls by all the three estimators (*P* = 0.08 for the Chao1 diversity index, *P* = 0.02 for the observed species diversity index, and *P* < 0.001 for the Shannon entropy index; by Wilcoxon rank-sum test, Fig. 2a).

We calculated the β-diversity of the samples using the weighted UniFrac distances and the Bray-Curtis dissimilarity to identify possible differences between the bacterial components in the gut microbiota of patients with PD and controls. The principal coordinates analysis (PCoA) revealed that the gut microbiota of patients with PD was distinct from those of the controls (*P* < 0.001 by a Permutational MANOVA (PERMANOVA) implementation using Uni-Frac distances and Bray-Curtis dissimilarity, Fig. 2b). These findings indicate that the richness and diversity of the gut microbiota in patients with PD are significantly different from that of controls.

### Alteration in gut microbiota between PD and control groups

**Fig. 3:**
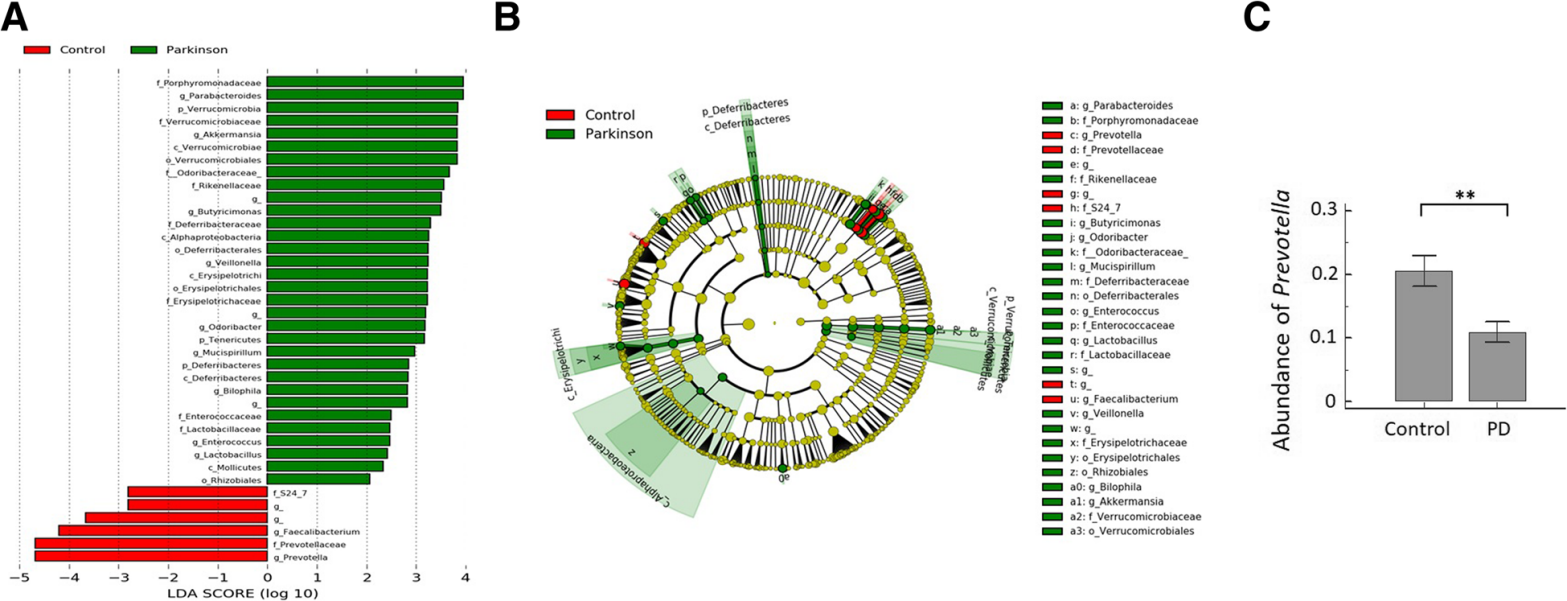
Taxonomic differences of fecal microbiota in PD and control groups. **a** Linear discriminant analysis (LDA) effect size (LEfSe) analysis revealed significant bacterial differences in fecal microbiota between the PD (positive score) and control groups (negative score). LDA scores (log10) > 2 and *P* < 0.05 are shown. **b** Cladogram using the LEfSe method indicating the phylogenetic distribution of fecal microbiota associated with PD and control participants. **c** The relative abundance of *Prevotella* was significantly higher in the control group than in patients with PD. **P < 0.01

A supervised comparison of the microbiota between PD and control groups was performed by linear discriminant analysis (LDA) effect size (LEfSe) analysis without any adjustments, which is often used to identify the presence and effect size of region-specific OTUs among different groups (Additional file 1: Supplementary Methods) [19]. We used a logarithmic LDA score cutoff of 2.0 to identify important taxonomic differences between the PD and control groups and found a notable difference in fecal microbiota between the PD and control groups based on LDA LEfSe analysis (Fig. 3a). We observed that the relative abundance of the *Prevotella* genus was higher in the control group than in the PD group, while the relative abundances of *Parabacteroides*, *Verrucomicrobia*, *Akkermansia*, *Butyricimonas*, *Veillonella*, *Odoribacter*, *Mucispirillum*, *Bilophila*, *Enterococcus*, and *Lactobacillus* were higher in patients with PD than in controls (LDA score (log10) > 2, Fig. 3a, b). GLMs with negative binomial distribution for bacterial abundances defined by sequence counts were used to model genera that were significantly different between the two groups after controlling for possible confounding factors such as age, sex, and diet (the mean daily intake amount of protein, carbohydrates, total fat, and dietary fiber). Microbiome differences between the groups were associated with *Verrucomicrobia*, *Prevotella*, *Mucispirillum*, *Porphyromonas*, *Lactobacillus*, and *Parabacteroides* (*P* < 0.05, Additional file 2: Table S2), suggesting an association between these genera and PD. The mean abundance of *Prevotella* was reduced by 46.6% in patients with PD compared to controls (Fig. 3c). The other genera were more abundant in patients with PD than in controls, but the absolute differences between the groups were smaller than that observed for *Prevotella* (data not shown).

### Association between fecal microbiota and PD clinical characteristics

**Fig. 4:**
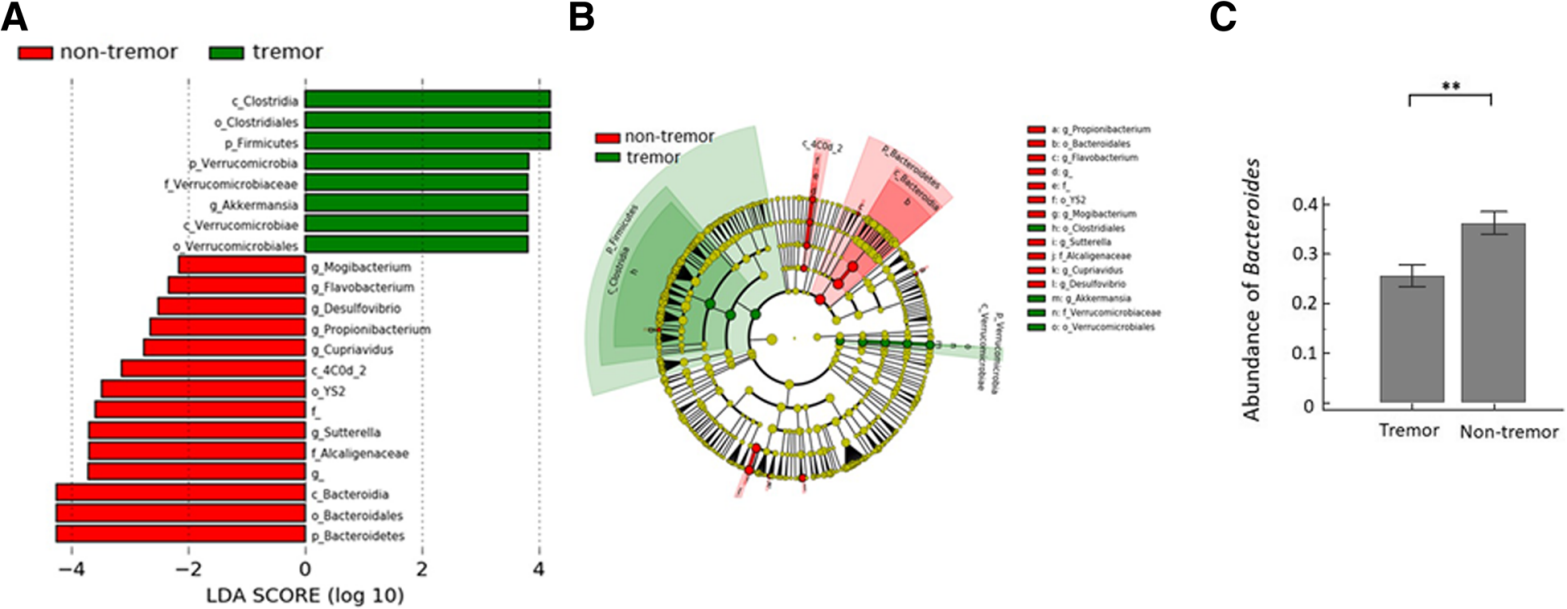
Taxonomic differences of fecal microbiota in tremor and non-tremor subtypes. **a** Linear discriminant analysis (LDA) effect size (LEfSe) analysis revealed significant bacterial differences in fecal microbiota between the tremor (positive score) and non-tremor subtypes (negative score) of PD patients. LDA scores (log10) > 2 and *P* < 0.05 are listed. **b** Cladogram using the LEfSe method indicating the phylogenetic distribution of fecal microbiota associated with tremor and non-tremor subtypes of PD patients. **c** The relative abundance of *Bacteroides* was significantly higher in the non-tremor group. **P < 0.01

We next examined the association between fecal microbiota and PD clinical subtypes. Of 80 PD patients, 47 (58.8%) were initially presented with tremor while the others presented with non-tremor subtypes, including the akinetic-rigidity or postural instability and gait difficulty (PIGD) subtypes. We found a significant difference in fecal microbiota between the tremor and non-tremor subtypes based on LDA LEfSe analysis without specific confounder adjustment (Fig. 4a). GLMs with negative binomial distribution for bacterial abundances defined by sequence counts were used to model genera that were significantly different between tremor and non-tremor subtypes after controlling for possible confounding factors such as age, sex, and diet (the mean daily intake amount of protein, carbohydrates, total fat, and dietary fiber). The relative abundances of *Clostridium*, *Verrucomicrobia*, and *Akkermansia* were higher in the tremor subtype than in the non-tremor subtypes, whereas the relative abundances of *Propionibacterium*, *Bacteroidia*, *Flavobacterium*, *Mogibacterium*, *Sutterella*, *Alcaligenacea Cupriavidus*, and *Desulfovibrio* were higher in the non-tremor subtype (LDA score (log10) > 2, Fig. 4a, b). Of these classes and genera, the mean abundance of *Bacteroides* genus from *Bacteroidia* class was increased by 41.6% in patients with non-tremor subtype PD compared to patients with the tremor-subtype (Fig. 4c). *Bacteroides* abundance also correlated with motor symptom severity as defined by UPDRS part III motor scores (rho = 0.637 [95% confidence interval 0.474 to 0.758], *P* < 0.01 by Spearman correlation analysis).

### Altered cytokine responses in PD patients with alterations in taxonomic compositions of the gut microbiota

**Fig. 5:**
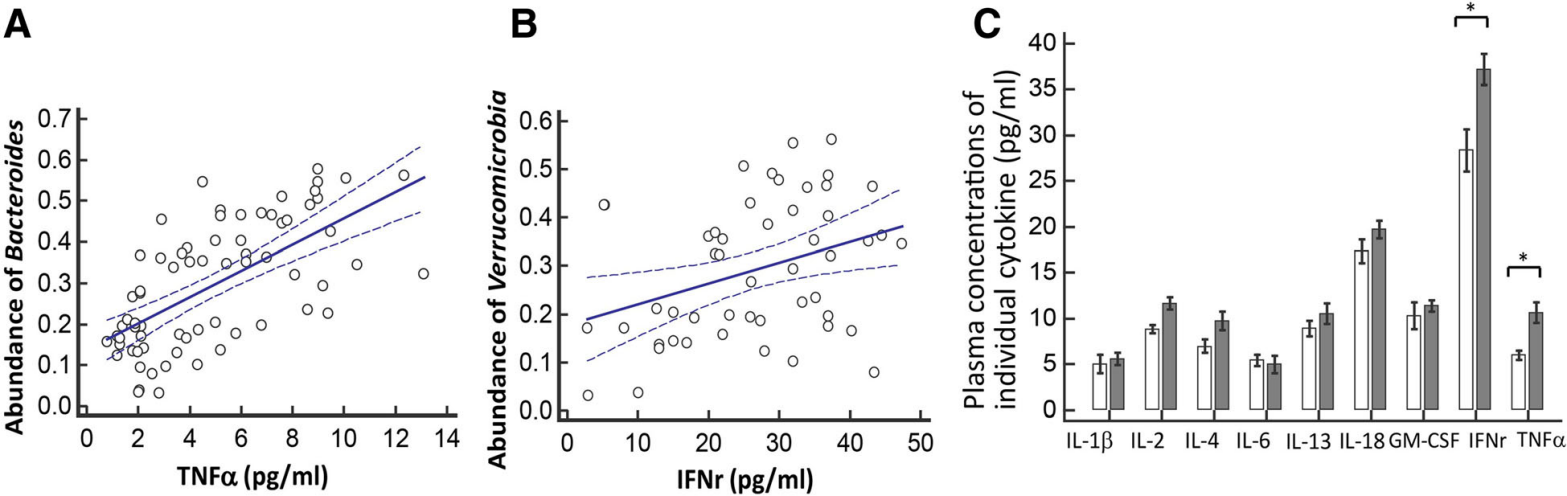
Correlations between abundances of specific fecal microbiota and plasma levels of cytokines. **a** The correlation between the relative abundances of *Bacterioides* and plasma concentration of TNFα. **b** The correlation between the relative abundances of *Verrucomicrobia* and plasma level of IFNγ. **c** Plasma concentrations of individual cytokines in PD and control groups in the firsts set of the study. The error bar indicated standard error of mean (SEM). PD, Parkinson’s disease. *P < 0.05. **P < 0.01

Because gut microbial dysbioses are often accompanied by abnormal production of inflammatory cytokines such as IL-1β, IL-22, and IFNγ [15, 20], we next investigated whether there are specific cytokine responses correlated with the relative abundances of the candidate genera associated with risk for or motor severity of PD, including *Verrucomicrobia*, *Prevotella*, *Mucispirillum*, *Porphyromonas*, *Lactobacillus*, *Parabacteroides*, and *Bacteroides.* Among the nine cytokines we examined in the human Th1/Th2 cytokine panel, we found there was a correlation between *Bacteroides* and plasma concentrations of TNFα (rho = 0.638 [95% CI 0.102–0.887], *P* = 0.02, Fig. 5a); and a correlation between *Verrucomicrobia* abundance and plasma concentrations of IFNγ (rho = 0.545 [95% CI − 0.043–0.852], *P* = 0.05, Fig. 5b). Consistently, the plasma levels of TNFα and IFNγ were increased in the PD group than controls (TNFα: PD vs. control: 10.71 ± 3.89 pg/ml vs. 6.04 ± 2.73 pg/ml, *P* = 0.02; and IFNγ: PD vs. control: 37.22 ± 9.15 pg/ml vs. 28.39 ± 10.21 pg/ml, *P* < 0.01) (Fig. 5c).

We then evaluated plasma cytokine responses in an additional independent 240 participants, including 120 patients with PD (62.3 ± 7.8 years, 62.5% men) and 120 age and sex-matched controls (61.8 ± 8.3 years, 60.0% men) to confirm the altered cytokine responses in the PD disease state. The plasma concentrations of TNFα and IFNγ were consistently and significantly higher in patients with PD than in controls (TNFα: PD vs. control: 8.51 ± 4.63 pg/ml vs. 4.82 ± 2.23 pg/ml, *P* < 0.01; and IFNγ: PD vs. control: 38.45 ± 7.12 pg/ml vs. 32.79 ± 8.03 pg/ml, *P* = 0.03; both were analyzed by one-way ANOVA) (Fig. 5). Additionally, the plasma level of IL-13 showed a marked increment in PD compared to the control group (12.49 ± 6.55 pg/ml vs. 2.93 ± 0.52 pg/ml, *P* < 0.01; by one-way ANOVA, Additional file 3). These results suggest that the altered microbiota seen in patients with PD may be associated with systemic inflammatory responses that contribute to PD development (Additional file 3).

## Discussion

Our study identified a clear gut microbiome composition alteration in individuals affected by PD in a large sample size recruited from an Asian population. The relative abundances of *Verrucomicrobia* and *Bacteroides*, which was either increased in PD patients or associated with motor symptom severity, correlated with elevated plasma concentrations of TNFα and IFNγ in patients with PD suggesting the occurrence of a systemic sub-inflammatory status associated with altered gut microbiota. Our results not only demonstrate that specific genera of gut microbiota may associate with the risk and severity of PD in our population, but also extend the current knowledge to glimpse the complexity of the interaction between gut microbiome composition alterations and host immune responses in PD pathogenesis.

We show that the richness and diversity of fecal microbiota as defined by α-diversity indexes were altered in patients with PD compared to those in the control group, consistent with previous reports from the USA [9] as well as another independent Chinese population [21]. The microbiome structures defined by the β-diversity index were qualitatively different between the PD and control groups. These observations were consistent with previous evidence suggesting that gut microbiota is altered in patients with PD [5, 8–10, 21, 22]. Although diets and ethnic backgrounds were distinct between Asian and Western populations, we identified some common genera changing in abundance in PD patients relative to controls. Our study observed that *Prevotella* abundance was reduced in patients with PD, in agreement with previous studies conducted in Germany, Finland, Russia, and Japan [5, 8, 10, 23, 24]. Trends toward a decrease in *Prevotellacea* were also reported in one study investigating the microbiome of the colonic mucosa rather than feces in PD patients [9]. *Prevotella* is a commensal microbe in the colon that maintains mucin glycoproteins in the gut mucosal layer but may also interact with the immune system [25]. Although some emerging studies in humans have linked the increased abundance of *Prevotella* in other systemic diseases, including periodontitis, bacterial vaginosis, rheumatoid arthritis, metabolic disorders, and low-grade systemic inflammation, a decreased *Prevotella* abundance is consistently observed in PD patients [5, 8–10, 23, 24]. The decreased *Prevotella* abundance is consistent with previous observations of increased gut permeability in PD, since low *Prevotella* levels may indicate decreased mucin synthesis, which is associated with leaky gut and aberrant gut immune responses and can lead to neurodegeneration in PD [7]. Decreased *Prevotella* has also been linked to reduced ghrelin concentration, and altered ghrelin secretion was reported previously in one PD study [5]. Together, our results suggest a possible protective role for *Prevotella* against neurodegenerative processes in PD. However, due to the conflicting role of *Prevotella* in other systemic medical disorders, the role of this genus in PD pathogenesis should be investigated further.

The relative abundance of the *Bacteroides* genus was higher in patients with the non-tremor PD subtype and correlated with motor symptom severity as defined by UPDRS part III motor sub-scores. Since PD is a clinically heterogeneous disorder, patients with the non-tremor subtype progress faster and show more severe α-synuclein pathology in colonic ENS neurons than patients with tremor [26]. The *Bacteroidetes* constitutes the largest phylum of Gram-negative bacteria in the gastrointestinal tract microbiome and some genera of *Bacteroidetes*, such as *Bacteroides fragilis*, has the potential to secrete a remarkably complex array of pro-inflammatory neurotoxins including surface lipopolysaccharides (LPSs) and toxic proteolytic peptides [27]. One recent microbiota analysis in fecal and colonic mucosa samples from patients with multiple system atrophy, another neurodegenerative disorder involving the nigra-striatal system, also revealed a relatively high abundance of *Bacteroidetes* [28]. We further identified a positive correlation between the abundance of *Bacteroides* genus from *Bacteroidetes* phylum and the plasma concentration of the pro-inflammatory cytokine TNFα in our PD patients. *Bacteroides* species have been shown to stimulate macrophages and monocytes to secrete TNFα by LPS-mediated pathways [29]. Consistently, one recent study identified elevated stool inflammatory profiles, such as interleukin-1 and CXCL8, in patients with PD compared to controls [30]. Future studies are needed to uncover the interactions between commensal gut microbiota changes and the immune reactions in PD pathogenesis.

We also found increased relative abundances of *Verrucomicrobia*, *Mucispirillum*, *Porphyromonas*, *Lactobacillus*, and *Parabacteroides* in the fecal microbiomes of patients with PD than in those of controls after adjusting for age and gender, consistent with previous reports of increased *Verrucomicrobia* and *Lactobacillus* abundance in patients with PD with diverse ethnicities [8, 11, 21, 22]. We also identified a modest correlation between *Verrucomicrobia* abundance and plasma concentrations of IFNγ, a pro-inflammatory cytokine produced by type I helper T cells [31]. Because altered gut microbiota are linked to aberrant gut and systemic immune responses, often accompanied by the abnormal production of inflammatory cytokines in the blood, our observations support a previous study showing that gut microbiota promoted motor deficits and neurodegeneration by activating neuroinflammation in an α-synuclein-overexpressing PD mouse model [4]. Additional functional studies are needed to elucidate the relationship between altered gut microbiome compositions and the immune reaction in the pathogenesis and progression of PD.

The major advantage of this study is the relatively large sample size from an East Asian population. Additionally, we correlate specific genera within fecal microbiota with plasma levels of pro-inflammatory cytokines and confirm the role of these cytokines in a large independent large cohort of patients with PD and controls. Our study also has some limitations. First, we did not check fecal short-chain fatty acid (SCFA) levels, which are a potential mediator of neurodegeneration caused by gut microbiome composition alterations [4], although the vast majority of studies suggest SCFAs play a beneficial and anti-inflammatory role in neurodegeneration [32]. A shotgun metagenome analysis could provide more detailed information about the microbiota, including the functional analysis, to analyze fecal microbiota in PD. Second, our study is a cross-sectional design, and we only considered diabetes mellitus and hypertension as major medical co-comorbidities in our study population. As many other comorbidities, for example, anxiety, depression, constipation, and other medical disorder, including rheumatoid arthritis and metabolic disorders, would also affect the gut microbiome, a longitudinal follow-up study considering all possible medical comorbidities and non-motor symptoms of PD will clarify the role of altered gut microbiota in disease progression. Finally, we did not analyze the correlations between every taxon and every cytokine listed in the panel due to the consideration of limited statistical power of multiple comparisons in the current study scale. We therefore only examined the taxa which was different between PD and controls to see the related changes of plasma cytokines under the hypothesis that altered microbiota may trigger systemic inflammatory responses contributing to PD. Future studies enrolling more participants and transcriptome assay to evaluate any possible changes of cytokines are needed to systemically explore the specific immune responses triggered by a particular genus or group of gut microorganisms.

## Conclusions

We find that gut microbiota is altered in PD and correlate with clinical phenotypes and disease severity in our population. The altered plasma cytokine concentrations associated with specific changes in microbiota further demonstrate that altered microbiome composition alterations with aberrant host immune responses are linked with PD pathogenesis. Elucidating the interplay between microbial and host immune responses will lead to a better understanding of PD pathogenesis.

## Additional files

Additional file 1 : Supplementary methods. (DOCX 21 kb)

Additional file 2 : Table S1 Clinical characteristics of the study participants. Table S2 General linear models for fecal genera based on differences between patients with PD and healthy controls. (DOCX 19 kb)

Additional file 3 : Figure S1 The plasma levels of individual cytokines in the second set of the study group. The plasma concentrations of IL-13, IFNγ, and TNFα were significantly higher in PD patients compared to control participants in the second set of the study design. The error bar indicated standard error of mean (SEM). PD: Parkinson’s disease. *P < 0.05. **P < 0.01. (JPG 127 kb)

## Data Availability

All data are available under request. The datasets used and analyzed during the current study are available from the corresponding author on reasonable request.

## Abbreviations

16S rRNA: 16S ribosomal RNA
ENS: Enteric nervous system
LB: Lewy bodies
LRRK2: Leucine-rich repeat kinase 2
MMSE: Mini-Mental State Examination
NMSS: Non-Motor Symptom assessment Scale
OTUs: Operational taxonomic units
PD: Parkinson’s disease
PIGD: Postural instability and gait difficulty
UPDRS: Unified Parkinson’s Disease Rating Scale
