# Supplementary material for: Altered gut microbiota and inflammatory cytokine responses in patients with Parkinson’s disease
Source: J Neuroinflammation. 2019 Jun 27;16:129. doi: 10.1186/s12974-019-1528-y (PMC6598278; doi:10.1186/s12974-019-1528-y)
Supplement: Supplementary file 1 — : Supplementary methods. (DOCX 21 kb) [file 12974_2019_1528_MOESM1_ESM.docx]

**Supplementary Methods:**

**PCR amplification of the V3-V4 region of bacterial 16S rRNA for library preparation**

Total DNA extraction from fecal samples (250 mg, wet weight) was performed using the QIAamp DNA Stool Mini Kit (Qiagen, Hilden, Germany) following the manufacturer’s instructions. DNA integrity and quality were checked on 1% agarose gel TAE 1X and quantified with a NanoDrop® spectrophotometer.

The 16S metagenomic sequencing libraries were generated according to the manufacturer's instructions as provided by Illumina (#15044223 Rev. B). Briefly, 12.5ng DNA was used for PCR amplification of the V3 and V4 regions of 16S rRNA genes. The PCR primers contain overhang adapter sequence and the full length primer sequences are as follows:

5’-TCGTCGGCAGCGTCAGATGTGTATAAGAGACAGCCTACGGGNGGCWGCAG (Forward primer) and

5'-GTCTCGTGGGCTCGGAGATGTGTATAAGAGACAGGACTACHVGGGTATCTAATCC (Reverse primer).

The PCR reaction mix contained 1X KAPA HiFi HotStart ReadyMix PCR buffer (Kapa Biosystems), 2 mM MgCl2, 200 μM of dNTPs, 0.4 μM of each primer, and 12.5 ng of template gDNA. The thermal cycling conditions were 3 min at 95 °C, 25 cycles of 30 s at 95°C, 30 s at 55°C and 30 s at 72°C followed by a final extension of 5 min at 72°C. All PCR experiments were carried out in triplicate using a Veriti® Thermal Cycler (Applied Biosystems, Foster City, CA, USA).

PCR products with the length of ~550bps were purified with AMPure XP beads (Beckman Coulter, USA) and subjected to a secondary PCR reaction with primers from the Nextera XT Index kit (Illumina, USA) which attaches the dual indices and Illumina sequencing adapters onto the V3 and V4 regions. The final libraries (~630bps) were purified after the PCR reaction with AMPure XP beads and were ready for next generation sequencing.

**Miseq-based high throughput sequencing and data analysis**

The concentrations of V3-V4 sequencing libraries of 16S rRNA were determined by Real-time quantitative PCR with Illumina adapter-specific primers provided by the KAPA library quantification kit (KAPA Biosystems, USA). Libraries were denatured and sequenced by the Illumina Miseq platform with V3 reagent for pair-end sequencing (2*300bps). Instrument controls, cluster generation, image capture, and base calling were processed by Real Time Analysis software (RTA) 1.18.54, MiSeq Control software (MCS) 2.5.0.5 and Miseq Report software 2.5.1.3 on the Miseq platform. FASTQ files generated by Miseq Report were used for further analysis. The raw sequences were denoised and quality filtered, before clustering into operational taxonomic units (OTUs) using a ≥97% sequence homology cutoff, and different phylogeny classes (i.e., Phylum, Class, Order, Family and Genus) based on GreenGenes reference taxonomy. Taxonomic analysis was performed by Quantitative Insights Into Microbial Ecology (QIIME) 1.9.1 and the classification was based on the Greengenes gg_13_8 database (<http://greengenes.lbl.gov/>). Alpha- (within-sample richness) and beta-diversity (between-sample dissimilarity) estimates were computed using the phyloseq R package [1]. Multiple-rarefaction PCoA plots (“jackknifed” PCoA plots, [2]) were computed to assess the robustness of the beta-diversity analyses. Permutational MANOVA (PERMANOVA) was performed on the Uni-Frac distances and Bray-Curtis dissimilarity using the adonis() function of the vegan R package with 999 permutations and without adjustment for specific confounders, and p values were corrected using the Bonferroni correction. Linear discriminant effect size analysis (LEfSe) [3] (http://huttenhower.sph.harvard.edu/lefse/) with an alpha cutoff of 0.05 and an effect size cutoff of 2.0 was performed to find taxonomic clades differentially represented between PD patients and controls. LEfSe combines Kruskal-Wallis test and Wilcoxon rank-sum tests with linear discriminant analysis (LDA). LEfSe ranks features by effect size, putting features that explain most of the biological difference at the top.

**Referrences of supplementary methods:**

1. Lozupone C, Lladser ME, Knights D, Stombaugh J, Knight R. UniFrac: an effective distance metric for microbial community comparison. ISME J. 2011;5(2):169–72.

2. Segata N, Izard J, Waldron L, Gevers D, Miropolsky L, Garrett WS,

Huttenhower C. Metagenomic biomarker discovery and explanation.

Genome Biol. 2011;12(6):R60.

3. McMurdie PJ, Holmes S. phyloseq: an R package for reproducible interactive

analysis and graphics of microbiome census data. PLoS One. 2013;8(4):e61217.
