# Supplementary material for: Altered gut microbiota and inflammatory cytokine responses in patients with Parkinson’s disease
Source: J Neuroinflammation. 2019 Jun 27;16:129. doi: 10.1186/s12974-019-1528-y (PMC6598278; doi:10.1186/s12974-019-1528-y)
Supplement: Supplementary file 2 — : Table S1 Clinical characteristics of the study participants. Table S2 General linear models for fecal genera based on differences between patients with PD and healthy controls. (DOCX 19 kb) [file 12974_2019_1528_MOESM2_ESM.docx]

**Supplementary Table 1. Clinical characteristics of the study participants.**

|  | Control  (n=77) | PD  (n=80) | *P* Value |
| --- | --- | --- | --- |
| Age (years) | 62.1±4.7 | 64.0±8.8 | *P*=0.12 |
| Gender (Male, %) | 46.7 | 53.6 | *P*=0.39 |
| Constipation (n, %) | 10 (12.3%) | 53 (66.3%) | *P<*0.01 |
| Disease duration (years) | N.A. | 7.5±5.2 |  |
| Motor symptom severity |  |  |  |
| Hoehn-Yahr stage (on) | N.A. | 1.8±0.9 |  |
| Hoehn-Yahr stage (off) | N.A. | 2.6±1.1 |  |
| UPDRS part III (on) | N.A. | 17.4±9.6 |  |
| UPDRS part III (off) | N.A. | 32.4±13.9 |  |
| Non-motor symptom severity |  |  |  |
| Overall NMSS scores (on) | N.A. | 32.4±13.9 |  |
| Medications for PD (n, %) |  | 80 (100%) |  |
| Levodopa | N.A. | 75 (93.4%) |  |
| Dopamine agonist | N.A. | 65 (81.3%) |  |
| MAO-B inhibitor | N.A. | 8 (10.0%) |  |
| COMT inhibitor | N.A. | 15 (18.8%) |  |
| Anticholinergics | N.A. | 10 (12.5%) |  |
| Amatadine | N.A. | 12 (15.0%) |  |
| LEDD (mg/day) | N.A. | 427.4 ± 259.9 |  |
| Medical Co-morbidities |  |  |  |
| Diabetes Mellitus (%) | 15.6 | 11.3 | *P*=0.09 |
| Hypertension (%) | 14.3 | 7.5 | *P<*0.01** |
| Main Diet Components |  |  |  |
| Protein (g/day) | 54.3±4.7 | 52.8±5.1 | *P*=0.17 |
| Carbohydrates (g/day) | 265.2±92.3 | 254.9±101.3 | *P*=0.10 |
| Total fat (g/day) | 44.6±3.6 | 42±4.1 | *P*=0.23 |
| Dietary fiber (g/day) | 22.8±1.8 | 24.7±3.1 | *P*=0.09 |

PD, Parkinson’s disease; UPDRS, unified Parkinson’s disease rating scale; NMSS, Non-Motor Symptom assessment scale; LEDD, levodopa equivalent dose; N.A., not available. Numbers are expressed as mean ± standard deviation. **P*<0.05, ** *P*<0.01.

**Supplementary Table 2. General linear models for fecal genera based on differences between patients with PD and healthy controls.**

| Genus | Odds ratio | 95% confidence interval | *P* value |
| --- | --- | --- | --- |
| Verrucomicrobia | 1.8241 | 0.0162-7.1852 | 0.0314 |
| Prevotella | -0.0595 | -0.0099 - -0.3580 | 0.00211 |
| Mucispirillum | 1.1177 | 0.005757-10.1694 | 0.01226 |
| Porphyromonas | 2.2150 | 0.002817-9.2671 | 0.01105 |
| Lactobacillus | 1.6660 | 0.00421-4.25672 | <0.0001 |
| Parabacteroides | 0.1028 | 0.00013-0.82142 | <0.0001 |

Result of the general linear models (GLMs) for significant genera (sequence counts) based on the group factors (PD and control group) and possible confounding factors (age, gender, diet) of 157 study participants. GLM, general linear model; CI, confidence interval.
