# Supplementary figures and images for: Altered gut microbiota and inflammatory cytokine responses in patients with Parkinson’s disease
Source: J Neuroinflammation. 2019 Jun 27;16:129. doi: 10.1186/s12974-019-1528-y (PMC6598278; doi:10.1186/s12974-019-1528-y)

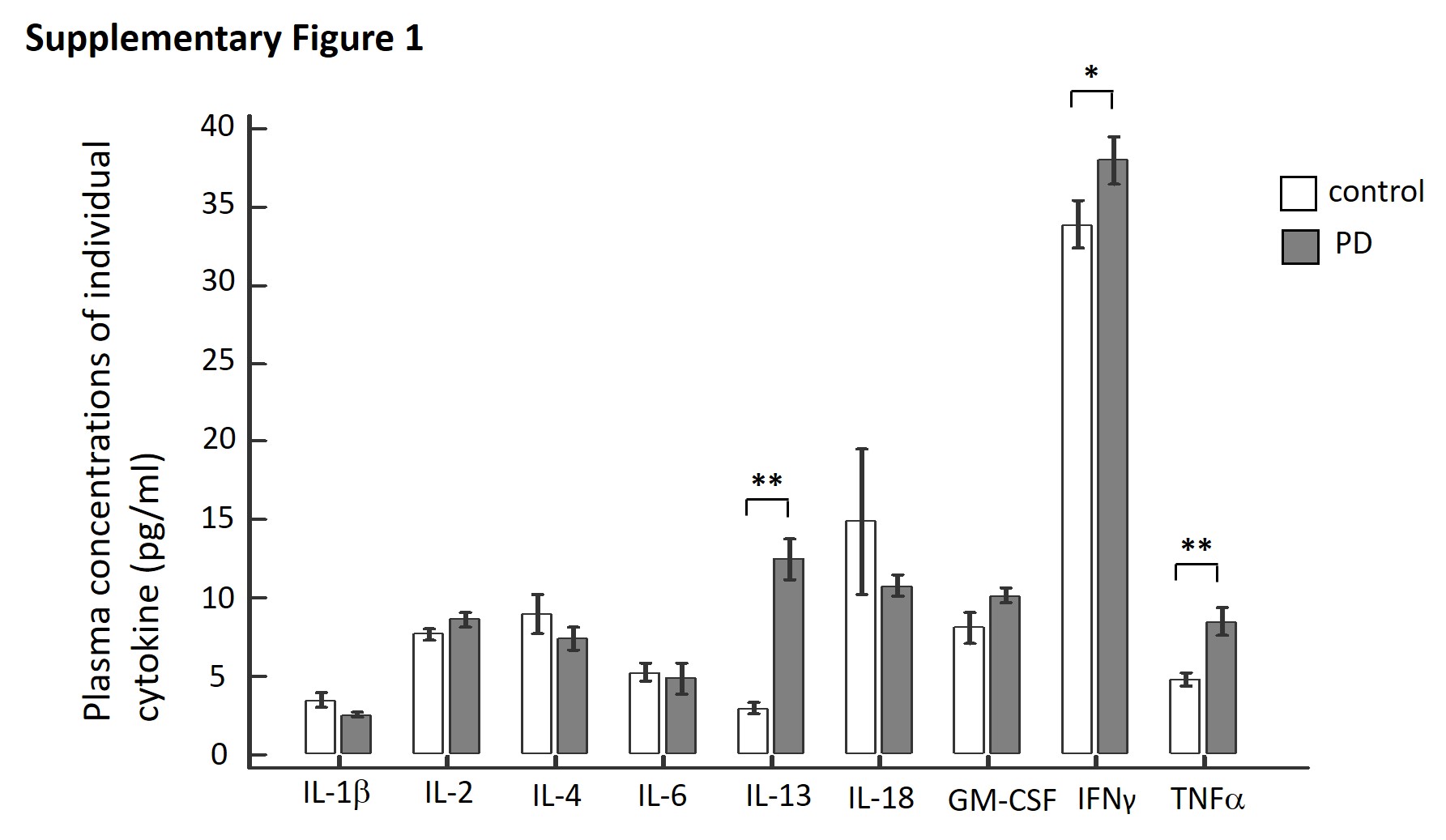

Supplement: Supplementary file 3 — : Figure S1 The plasma levels of individual cytokines in the second set of the study group. The plasma concentrations of IL-13, IFNγ, and TNFα were significantly higher in PD patients compared to control participants in the second set of the study design. The error bar indicated standard error of mean (SEM). PD: Parkinson’s disease. *P < 0.05. **P < 0.01. (JPG 127 kb) [file 12974_2019_1528_MOESM3_ESM.jpg]
